# Detection of antibiotic resistant Enterobacterales in commercial raw pet food: a preliminary study

**DOI:** 10.3389/fvets.2024.1294575

**Published:** 2024-06-12

**Authors:** Carolyn D. Fisher, Douglas R. Call, Sylvia Omulo

**Affiliations:** ^1^Paul G. Allen School for Global Health, Washington State University, Pullman, WA, United States; ^2^Washington State University Global Health–Kenya, Nairobi, Kenya; ^3^University of Nairobi Institute of Tropical and Infectious Diseases, Nairobi, Kenya

**Keywords:** extended-spectrum beta-lactam-resistance, ESBL, Enterobacterales, pet food, raw diet, RMBD

## Abstract

**Introduction:**

Raw diets have become popular in companion animal nutrition, but these diets may be contaminated with harmful bacteria because heat processing is not utilized to mitigate pathogens during the production process. We analyzed 24 commercially available frozen raw canine and feline diets for extended-spectrum beta-lactamase-producing Enterobacterales (ESBL-E).

**Methods:**

Samples were incubated in tryptic soy broth augmented with 50 μg/mL ampicillin to enrich for ESBL-E. ESBL-E were isolated using CHROMagar ESBL plates and isolate identification and antibiotic susceptibility testing were confirmed using the VITEK^®^2 instrument.

**Results:**

ESBL-E were isolated from 42% (10/24) of raw diets, with *E. coli, Enterobacter cloacae* complex and *Klebsiella pneumoniae* predominating. Most ESBL-E isolates (71%, 32/45) were multidrug-resistant. Direct plating of samples onto tryptic soy agar yielded bacterial counts >6 log_10_ for 2 samples from two different manufacturers.

**Conclusion:**

This preliminary study justifies further investigation into the potential contribution of raw diets to the dissemination of antibiotic resistant bacteria in companion animals and domestic living spaces.

## Introduction

A growing number of pet owners are choosing raw meat-based diets (RMBDs), also known as “raw diets,” for their pets (1–3). RMBDs are formulated using uncooked ingredients including muscle, organ meat and bone sourced from domestic and wild animals (1). Pet owners often elect to feed RMBDs due to mistrust of conventional diets and the belief that RMBDs are healthier and more natural diets for pets (4–6). There are perceived health benefits to feeding RMBDs, including improved dental health, coat quality and muscle mass. However, these claims are unsubstantiated by current literature (1, 5, 6). Approximately 25% of North American agility dog owners feed their dogs RMBDs and a 2020 survey found that 9% of pet owners across Canada, New Zealand, Australia, and the US feed RMBDs exclusively (2, 3). Notably, the Centers for Disease Control and Prevention, the American Veterinary Medical Association, and the U.S. Food and Drug Administration advise against RMBDs due to the risk of bacterial contamination and transmission to pets and owners (7–9). Raw meat can become contaminated with pathogens during slaughter, processing and transportation and there is existing evidence of RMBD cross-contamination during manufacturing (1, 6, 10). Previous studies have detected DNA from undeclared protein sources in RMBDs and genetically-identical bacteria has been isolated from RMBDs with different protein sources that were produced by the same manufacturer (10, 11). *Salmonella* species are often a prominent concern, as these bacteria cause 26,500 hospitalizations in the United States annually (12). Furthermore, the risk of bacterial contamination is made more serious by the threat of antimicrobial resistance. In 2019, antimicrobial-resistant bacteria contributed to an estimated 4.95 million human deaths globally (13). Extended-spectrum β-lactamase-producing Enterobacterales (ESBL-E) are especially concerning and have a public health importance because they can inactivate critical β-lactam antibiotics such as cephalosporins and carbapenems. In 2017, ESBL-E were responsible for 9,100 estimated deaths in the United States (14).

The extent to which RMBDs disseminate ESBL-E remains largely unknown (15). Studies in Europe have isolated ESBL-E from 61% (31/51) of sampled RMBDs and ESBL-producing *Escherichia coli* from 80% (28/35) of sampled RMBDs (16, 17). Additionally, a 2017 study in the Netherlands detected ESBL-E in 78% (14/18) of RMBD products but none in non-raw pet foods (18). Data on ESBL-E contamination of RMBDs in the United States are scant; a recent study isolated ESBL-E from 10% (20/200) of RMBD products (11). These findings indicate the potential public health threat posed by RMBDs in the prevailing antibiotic resistance crisis. We analyzed commercially available frozen RMBDs in the US to assess ESBL-E contamination, examine the antibiotic resistance profiles of ESBL-E isolates and characterize aerobic bacterial contamination of RMBDs.

## Methods

### Product selection

For this preliminary study, a convenience sample of RMBDs that met the following criteria were selected: (i) frozen products intended for consumption by dogs, or dogs and cats, (ii) products containing a single animal protein and whose meat was sourced from the US, and (iii) products that were not freeze-dried, pasteurized, fermented, or high pressure processed. RMBDs were either purchased online and shipped via ground transport from the distributor, or from a retail store and transported to the laboratory within 3 h of purchase. Transportation was done under chilled conditions. Upon arrival, each diet was photographed, and the integrity of packaging was assessed. Diets were immediately placed in a −20°C freezer. Lot numbers and expiration dates were recorded where available.

### Sample processing

Each product was stored in a −20°C freezer for a median of 18 days (range: 1–38 days) in its original packaging. Prior to processing, the products were thawed at 4°C for 26–28 h and transferred into sterile 24 oz Whirl Pak sample bags (Fort Atkinson, WA) in a biosafety cabinet. The products were homogenized by hand for 60 s. Six 2-g samples were collected from each raw diet for processing in duplicates; three samples were added to 10-ml tryptic soy broth (TSB) augmented with ampicillin (50 μg/ml) and three samples to 10-ml of sterile water to enumerate total bacterial counts (colony forming units; CFUs). Each sample was assigned a unique laboratory identification code based upon the food brand, protein source and order that the samples were collected from the homogenized diet.

The TSB tubes were mixed for 30 s using a vortex to form slurries, then incubated in a 37°C shaker overnight (18–24 h). Ten microliters (10 μl) of the resulting TSB-ampicillin cultures were streaked onto CHROMagar ESBL plates (CHROMagar, Paris, France). The plates were incubated at 37°C overnight. Blue colonies on ESBL plates—presumptively identified as *Klebsiella, Citrobacter*, or *Enterobacter* sp.—or pink—presumptively *E. coli*—were collected from each positive plate and purified on trypticase soy agar (TSA) plates. A maximum of four colonies of different colors and morphologies were selected from a single ESBL plate for purification on a TSA plate. After overnight incubation at 37°C, one colony from each TSA plate was transferred to TSB broth with 50% v/v sterile glycerol and archived in a −80°C freezer. Archived isolates were revived by streaking onto TSA plates and incubating at 37°C overnight. One colony from each TSA plate was re-streaked for isolation on a second plate prior to identification and antibiotic susceptibility testing using VITEK^®^2 GN ID and AST GN84 cards, respectively. The GN84 card was selected for its ESBL confirmatory test and inclusion of antibiotics from three most frequently used antibiotic classes in food animals (19). Breakpoints for each antibiotic and bacterial species followed CLSI M100 (20). ATCC-BAA-2469 was a positive control for ESBL plates while ATCC 2912 and ATCC 25922 were negative controls. All controls were also used with the VITEK^®^2. Isolates were tested for susceptibility to 16 antibiotics belonging to nine antibiotic classes, including aminoglycosides, carbapenems, cephalosporins, fluoroquinolones, monobactams, nitrofurans, penicillins, sulfonamides and tetracyclines.

The sterile water slurries were used for CFU counts. Ten-fold serial dilutions (up to 10^−5^) of slurries were prepared using 0.9% sterile saline in a 96-well plate. Five microliters (5 μl) of each dilution were transferred onto TSA plates using a multi-channel pipette. The plates were inverted and incubated at 37°C for 16–18 h. CFU counts were calculated from dilutions containing 25–250 discrete colonies.

### Data analysis

Isolates of the same bacterial species with identical antibiotic resistance phenotypes that were recovered from the same initial 2-g raw food sample were assumed to be duplicates. Multidrug resistance was defined as resistance to at least one antibiotic from ≥3 antibiotic classes (21). Isolates with intermediate resistance were classified as susceptible. Data entry and summaries of the antimicrobial resistance profiles, recovered bacterial species, protein sources, lot numbers and expiration dates were performed in Microsoft Excel. Data analysis was limited to descriptive data.

## Results

Twenty-four RMBDs from seven manufacturers were purchased between May and July 2021. Between 2–5 RMBDs were evaluated from each manufacturer, with a mode of three products per manufacturer. Fifty-eight percent (14/24) products were purchased online while 42% (10/24) were purchased in a retail store. Fifty-four percent (13/24) had lot numbers and 67% (16/24) had expiration or packaging dates. The RMBDs included five protein sources (beef, chicken, duck, lamb, and turkey). Forty-six percent (11/24) diets included vegetables in their ingredients, 42% (10/24) had meat only, while 4% (3/24) had no ingredients listed. One manufacturer (*n* = 4 products) used bacteriophages and blanched their vegetables as a food safety measure; ESBL-E were isolated from one product by this manufacturer.

ESBL-E were isolated from 42% (10/24) of the RMBDs representing 71% (5/7) manufacturers, with 56% (25/45) ESBL-E isolates sourced from a single manufacturer and 88% (39/45) ESBL-E isolates from three manufacturers. In terms of ingredients, ESBL-E were isolated from 27% (3/11) diets with meat and vegetables, 33% (3/10) diets with meat only, and 67% (2/3) diets with unlisted ingredients (Table 1). Of the five protein sources, ESBL-E were detected in 50% (3/6) of beef, 33% (2/6) of chicken, 25% (1/4) of lamb, 33% (1/3) of duck, and 60% (3/5) of turkey diets. Fifty-six presumptive ESBL-E isolates were analyzed using the VITEK^®^2. Of these, 93% (52/56) were confirmed ESBL-positive. After excluding six putative clonal isolates and an outlier *Citrobacter* sp. isolate, 45 ESBL-E isolates were analyzed. *E. coli* (42%, 19/45)*, Enterobacter cloacae* complex (36%, 16/45), and *Klebsiella pneumoniae* (22%, 10/45) were the predominant bacterial species. *E. coli* was the only ESBL-E isolated from chicken diets; 42% (8/19) of all *E. coli* isolates were from chicken (Table 1).

**Table 1:** Distribution of recovered ESBL-E bacteria by manufacturer, protein source, other ingredients, and bacterial species.

**Manufacturer ID**	**Protein source**	**Listed ingredients**	***E. cloacae* complex (*n* = 16)**	***Escherichia coli* (*n* = 19)**	***Klebsiella pneumoniae* (*n* = 10)**
A12	Beef	Meat and vegetables	4	0	0
A12	Turkey	Meat and vegetables	3	0	0
B10	Beef	–	0	5	3
B10	Chicken	Meat only	0	5	0
B10	Lamb	Meat only	0	2	2
B10	Turkey	Meat only	0	4	4
C19	Turkey	Meat only	2	0	1
K11	Beef	–	2	0	0
K11	Duck	Meat and vegetables	5	0	0
O16	Chicken	Meat and vegetables	0	3	0

CFU counts were possible for 42% (10/24) total diets. ESBL-E were detected in 33% (3/10) of these diets, including one turkey diet, one lamb diet with <50 CFUs, and one beef diet with 4.0 × 10^6^ CFUs. The remaining diets had CFU counts ranging from <50 CFUs/g to too numerous to count, consistent with significant bacterial contamination (Table 2).

**Table 2:** Mean CFU counts for 10 diets.

**Manufacturer ID**	**Protein**	**Listed ingredients**	**ESBL-E detected**	**Mean CFU/g**
A12	Beef	Meat and vegetables	Yes	4,000,000
A12	Chicken	Meat and vegetables	No	Numerous
A12	Turkey	Meat and vegetables	Yes	<50
B10	Duck	Unknown	No	290,000
B10	Lamb	Meat only	Yes	<50
D14	Beef	Meat and vegetables	No	260,000
D14	Chicken	Meat and vegetables	No	1,100,000
D14	Turkey	Meat and vegetables	No	440,000
P18	Beef	Meat and vegetables	No	<50
P18	Lamb	Meat and vegetables	No	280,000

**Figure 1:**
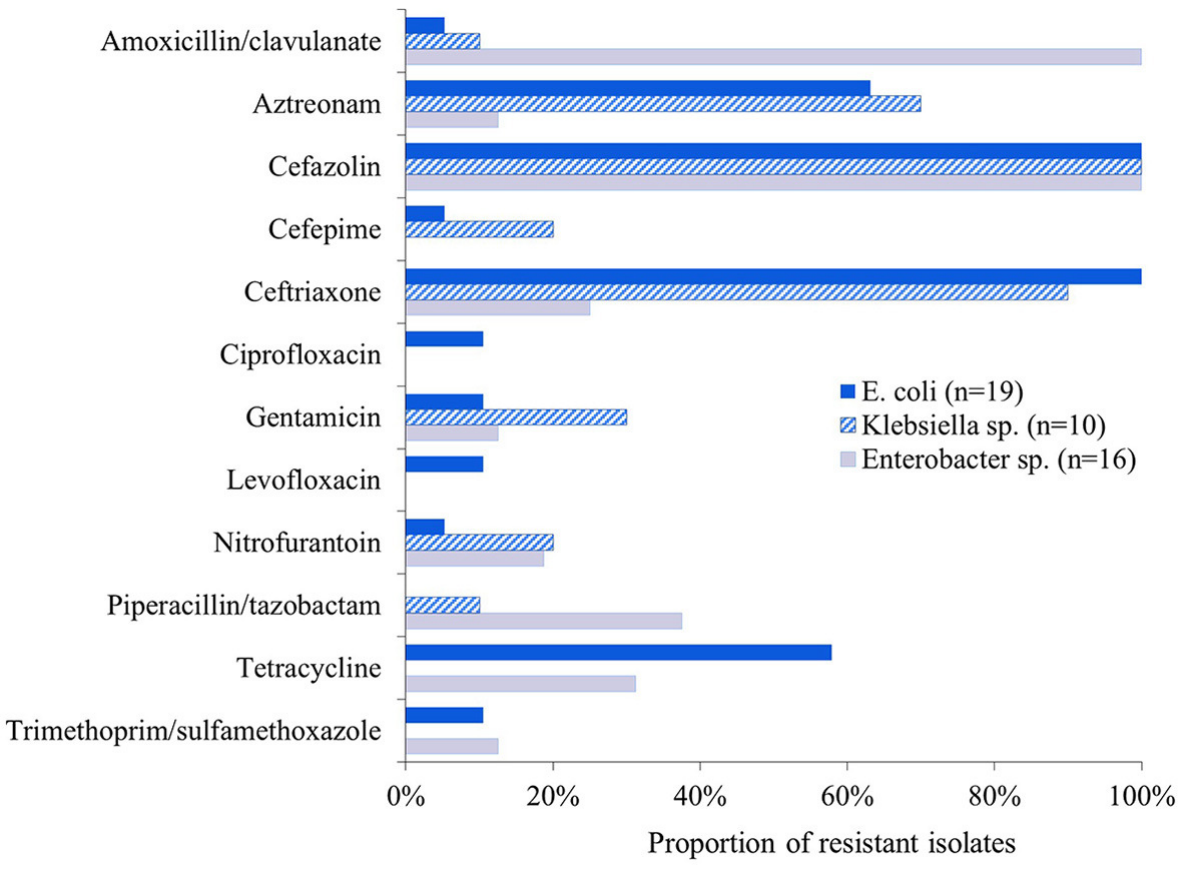
Proportion of ESBL-E isolates (*n* = 45) that were resistant to the 16 antibiotics tested. All isolates were susceptible to ertapenem, imipenem, and meropenem.

All isolates were resistant to cefazolin and susceptible to ertapenem, imipenem and meropenem. Forty per cent (18/45) were resistant to amoxicillin-clavulanic acid, including 100% (16/16) *Enterobacter* sp., 10% (1/10) *Klebsiella* sp. and 5% (1/19) *E. coli* isolates. Most isolates (71%, 32/45) were resistant to ceftriaxone and 47% (21/45) to aztreonam, with *Klebsiella* and *E. coli* predominating. Several isolates (36%, 16/45) were resistant to tetracycline with *E. coli* and *Enterobacter* predominating (Figure 1). Most ESBL-E isolates (71%, 32/45) were multidrug-resistant with 31% (14/45) being resistant to three antibiotic classes, and 29% (13/45), 7% (3/45), and 4% (4/45) being resistant to four, five, and six antibiotic classes, respectively.

## Discussion

In European studies, ESBL-E prevalence values of >60% have been reported while an American study found a prevalence of 10% in the sampled products (11, 16–18). Consequently, the isolation of ESBL-E in the present study was expected. Nevertheless, our study reported a higher prevalence (42%) than that previously reported in America, potentially due to disproportionate ESBL-E contamination among RMBD manufacturers. In our study, over half (56%) of ESBL-E isolates were detected in diets sourced from a single manufacturer. This is consistent with a study in which 75% of contaminated products came from 4/61 manufacturers; multiple products of different protein sources from the same manufacturers also contained genetically identical bacteria (11). Furthermore, a 2020 North American study recovered the DNA of at least one undeclared animal source in >60% of RMBDs analyzed (10). These results indicate that the manufacturing process and cross-contamination may significantly influence ESBL-E contamination of RMBDs.

ESBL-E have more frequently been isolated from frozen raw diets than freeze-dried or other raw diet preparations (11, 22). A previous American study recovered ESBL-E from frozen but not from freeze-dried raw diets (11). This difference may exist because the reduced water content in freeze-dried foods creates suboptimal conditions for bacterial survival (11, 23). It is also possible that different thawing methods may impact the quantity of bacteria recovered from frozen RMBDs. One study noted a significant increase in aerobic bacterial CFU counts 24 h after RMBD defrosting began, at 2 and 7°C (24). These results demonstrate the need for further research to elucidate the safest RMBD processing and feeding practices.

Several studies have isolated ESBL-producing *E. coli* from RMBDs (11, 16, 17), so it is not surprising to isolate *E. coli*. The proportions of *K. pneumoniae* (22%) and *E. cloacae* (35%) isolated in the present study were higher than previously reported (6%−10% and 0%−2%, respectively) (11, 16). *Enterobacter* sp. were isolated from three manufacturers and three distinct protein sources, indicating that *Enterobacter* sp. contamination was not unique to a single manufacturer or protein source. Conversely, *Klebsiella* was only isolated from two manufacturers, with 90% of the isolates recovered from a single manufacturer. It is possible that this disproportionate contamination is responsible for the higher overall prevalence of *Klebsiella* in our study.

The proportion of multi-drug resistant ESBL-E (71%) and pan-susceptibility to carbapenems observed in the present study were consistent with previous findings (11, 16). Previous literature from the Netherlands reported no ESBL-*E. coli* resistance to tetracycline (25), whereas our study found that 57% of *E. coli* isolates were resistant to tetracycline. This discrepancy is intriguing given that tetracyclines had the highest food animal antibiotic sales volume in both the Netherlands and United States when these studies were conducted (26, 27). With regards to sulfonamides, a Swiss study found that 50% of ESBL-E isolates were resistant to trimethoprim-sulfamethoxazole whereas only 9% were resistant in our study. This disparity may exist because sulfonamides had the second and third highest food animal antibiotic sales volume in the countries where RMBDs were sourced for the study in Switzerland (28, 29). In contrast, sulfonamides comprise 5% of medically-important food animal antibiotic sales in the U.S. (26). These results indicate the need to investigate how national antibiotic usage impacts antibiotic resistance phenotypes in RMBDs.

Data on bacterial contamination of RMBDs are unavailable in the US. Studies in Europe have reported aerobic mesophilic and Enterobacterales counts of 8.2 × 10^4^-7.4 × 10^8^ CFU/g, and aerobic bacterial counts of 7.9 × 10^2^-5.0 × 10^6^ CFU/g and 4.22 × 10^4^ CFU/g−3.77 × 10^6^ CFU/g (16, 17, 30). The counts of aerobic bacteria in the present study varied from <50 CFU/g to numerous. The quantifiable upper range (4.0 × 10^6^ CFU/g) is comparable to the upper range of aerobic bacteria in previous studies (5.0 × 10^6^ CFU/g and 3.77 × 10^6^ CFU/g) (17, 30). The wide range of contamination recorded in the present study suggests that further work is needed to identify sources of bacterial contamination of RMBDs.

RMBDs pose a potential risk to public health, as people can be exposed to harmful bacteria by handling contaminated diets or the feces of RMBD-fed pets (31). Pets fed RMBDs are more likely to shed harmful bacteria in their feces (18, 32–34); a 2022 study demonstrated clonal relationships between *Salmonella* sp. isolated from RMBDs and canine fecal samples from the same household (31). Moreover, a study in Brazil reported that dogs fed RMBDs were 30 times more likely to shed *Salmonella* in their feces compared to dogs fed commercial dry food (33). Similarly, a cohort study that compared RMBD-fed cats and those not fed RMBDs isolated ESBL-E in 90 and 6% of cat stool samples, respectively (18). Contaminated feces may pose health risks to children; a Canadian survey found that 52% of household reported that their children (<16 years) play in the same areas where their dogs defecate (35). While the risk of human infection with antibiotic-resistant bacteria shed by pets consuming RMBDs has not been reported, household transmission of antibiotic-resistant Enterobacterales between dogs and humans has been documented (36). A 2020 study in New Zealand found that in 22% of households, clonal strains of ESBL-producing *E. coli* were cultured from both a person and pet within the household (36). Likewise, a 2019 study in the Netherlands noted that “eating raw meat” was a predictor of ESBL-E carriage in canines and that human-canine ESBL-E co-carriage was higher than predicted based on chance (0.9%) (37). Although these studies show that the proportion of household human-canine co-carriage of ESBL bacteria is relatively small, 69 million households in the US own a dog and—based on a survey of 1,250 dog owners in the US-−63% included raw food as a part of their dog's diet (2, 38). Based on this data, even if the proportion of affected household is as low as 1%, potentially >400,000 American households could be affected. These results emphasize the need for long-term studies to establish the directionality of ESBL-E transfer between humans and pets, identify sources of ESBL-E, and further describe the persistence of ESBL-shedding in pets.

In the present study, 46% of RMBD samples did not include lot numbers and 33% did not have expiration or packaging dates. The FDA recommends including lot numbers on pet food for ease of recall and to facilitate the reporting of product concerns (39). It requires that pet food includes an ingredients list, nutritional adequacy statement and guaranteed analysis on packaging (22, 40, 41); 13% of samples representing two different brands did not meet any of these requirements. The nutritional adequacy statement is important as it indicates whether the diet will meet the pet's daily nutrient needs (41). Comparably, a study in Minnesota found that 27% of RMBD brands evaluated did not include a nutritional adequacy statement or guaranteed analysis (22). These results demonstrate that some RMBD manufacturers omit important information that could impact the health and safety of pets.

The present study was intended as a small-scale preliminary study and as such, there were several constraints. As we did not conduct whole genome sequencing, we were unable to confirm isolate uniqueness for data analysis or genetic variation of the ESBL-E recovered from RMBDs. These are potential research areas for future studies. Additionally, the relative abundance of ESBL-E organisms compared to normal flora in the RMBDs is unclear, as the CFU counts in the present study only allowed for a snapshot of overall microbiological contamination of the RMBDs. The present study was also limited to seven brands and 24 diets that could be purchased and delivered to Washington state in the US. It is possible that the samples analyzed in the present study were not representative of commercially available frozen RMBDs. The present study did not allow for an overall prevalence estimate of ESBL-E contamination of RMBDs in the US. Additional research is required to establish these prevalence estimates. Freeze-dried, pasteurized, high pressure processed and fermented RMBD preparations were not investigated in the present study. Further research is needed to elucidate the prevalence of ESBL-E in these RMBD preparations. The number of diets in the present study did not allow for a comparative analysis of contamination in RMBDs with different protein sources and ingredients. This is an area that future research could explore.

## Conclusion

This study advances our knowledge of the ESBL-E bacteria that humans and animals may encounter through RMBDs. This can guide the development of intervention strategies, educate veterinarians, and advise pet owners about the risks involved with feeding RMBDs. While more data are needed to establish the true prevalence of ESBL-E in frozen RMBDs in the U.S., this study and the previous literature emphasize the need for good hygiene practices when feeding RMBDs and handling pets that are fed RMBDs.

## Data availability statement

The raw data supporting the conclusions of this article will be made available by the authors, without undue reservation.

## Author contributions

CF: Conceptualization, Data curation, Formal analysis, Investigation, Methodology, Project administration, Writing – original draft. DC: Methodology, Project administration, Resources, Supervision, Writing – review & editing. SO: Formal analysis, Funding acquisition, Methodology, Resources, Supervision, Writing – review & editing.
